# Pain Inhibition by Optogenetic Activation of Specific Anterior Cingulate Cortical Neurons

**DOI:** 10.1371/journal.pone.0117746

**Published:** 2015-02-25

**Authors:** Ling Gu, Megan L. Uhelski, Sanjay Anand, Mario Romero-Ortega, Young-tae Kim, Perry N. Fuchs, Samarendra K. Mohanty

**Affiliations:** 1 Biophysics and Physiology Group, Department of Physics, University of Texas at Arlington, Arlington, TX-76019, United States of America; 2 Department of Psychology, University of Texas at Arlington, Arlington, TX-76019, United States of America; 3 Department of Bioengineering, University of Texas at Arlington, Arlington, TX-76019, United States of America; 4 Departments of Psychology and Biology, University of Texas at Arlington, Arlington, TX-76019, United States of America; University of Texas at Dallas, UNITED STATES

## Abstract

Cumulative evidence from both humans and animals suggests that the anterior cingulate cortex (ACC) is important for pain-related perception, and thus a likely target for pain relief therapy. However, use of existing electrode based ACC stimulation has not significantly reduced pain, at least in part due to the lack of specificity and likely co-activation of both excitatory and inhibitory neurons. Herein, we report a dramatic reduction of pain behavior in transgenic mice by optogenetic stimulation of the inhibitory neural circuitry of the ACC expressing channelrhodopsin-2. Electrophysiological measurements confirmed that stimulation of ACC inhibitory neurons is associated with decreased neural activity in the ACC. Further, a distinct optogenetic stimulation intensity and frequency-dependent inhibition of spiking activity in the ACC was observed. Moreover, we confirmed specific electrophysiological responses from different neuronal units in the thalamus, in response to particular types of painful stimuli (i,e., formalin injection, pinch), which we found to be modulated by optogenetic control of the ACC inhibitory neurons. These results underscore the inhibition of the ACC as a clinical alternative in inhibiting chronic pain, and leads to a better understanding of the pain processing circuitry of the cingulate cortex.

## Introduction

Chronic pain is a major world-wide health issue, leading to severe impairment of patient’s normal psychological and physical function [1]. Chronic pain is associated with long-term over-activity of sensory pathways involved in the natural processing of noxious information such as peripheral nociceptors, interneurons in the spinal dorsal horn, thalamic nuclei, and sensory cortex [2]. Sustained inhibition of such circuits has been proposed as a possible strategy to mitigate pain. However, inhibition of idiopathic chronic pain is rarely achieved, and management of chronic pain remains a significant challenge. Therefore, there is an intense need for increased understanding of pain pathways and the capability to selectively targeting neuronal systems that may inhibit the perception of chronic pain. Collective evidence from both human and animal studies suggests that the anterior cingulate cortex (ACC) is an important area for pain-related perception [3,4]. Electrophysiological recordings demonstrate that neurons within the ACC respond to noxious stimuli and are activated during pain anticipation [5,6,7]. In addition, activation of the ACC is associated with reduced pain behavior [8] and a reduction in spinal dorsal horn activity [9]. Neuroimaging studies further confirm that the ACC, together with other cortical structures, is activated by physical pain as well as “psychological” pain [10,11,12,13,14]. Our previous research has demonstrated differential effects of ACC lesions on mechanical hypersensitivity and escape/avoidance behavior in an animal model of neuropathic pain [15]. Others have reported that lesions of the ACC leads to significantly increase in nociceptive responses [16,17]. These controversial findings might be accounted for by function-specific sub-regions [18] and their connectivity, such as subgenual (for depression) and pregenual ACC (for cognitive regulation)[19]. However, it still remains to be determined if such regions exist within the ACC at the synaptic and molecular levels.

The advent of neuronal stimulation using optogenetics has enabled highly selective activation of several types of neurons with millisecond temporal precision [20,21]. As compared to electrical stimulation, optogenetics is more specific and multiple types of neurons can be selectively targeted within the same region of the nervous system [22,23]. This light-assisted method of cellular stimulation eliminates the highly challenging requirement of placing electrodes in brain nuclei with relatively homogeneous group of neurons. This characteristic has also led to the emerging of optogenetics as a valuable experimental tool and a promising approach for studying a variety of neurological disorders, such as blindness [24,25,26,27], drug-addiction [28,29], conditioned fear [30], and Parkinsonian symptoms [31] in animal models. Since channelrhodopsin-2 (ChR2), a non-selective cation channel, is the most commonly used opsin for depolarization of neurons [21,32], we chose to evaluate transgenic (Thy1-ChR2-YFP) mice for studying the effect of optogenetic stimulation of ACC on pain sensation.

Herein, we demonstrate that stimulation of inhibitory neurons in ACC by optogenetic means leads to decreased activity of the ACC and a significant reduction of pain perception of diverse types of noxious stimuli. Electrophysiological measurements show a distinct frequency-dependent inhibition of spiking activity in the ACC with optogenetic stimulation of inhibitory neurons. With this method, the electrophysiological responses of different neuronal units in the thalamus normally elicited in response to different types of pain including chemical (formalin injection), or mechanical (pinch and brush), were differentially modulated in response to stimulation of ACC expressing ChR2 in inhibitory neurons. These results open up a new avenue to the understanding of the pain processing circuitry of the cingulate cortex and support the use of optical inhibition of ACC as possible treatment for chronic pain.

## Material and Methods

### Ethics statement

All experimental procedures were conducted according to the University of Texas at Arlington-Institutional Animal Care and Use Committee approved protocol A10.009. The IACUC specifically approved this study.

### Determination of light propagation for controlled stimulation of ACC

In order to determine the parameters for light delivery (e.g. intensity) to the ACC in a controlled manner, Monte Carlo (MC) simulation software (BeamMCML) was developed based on the widely used MCML software [33], which is capable of simulating light in multi-layered media. First, collimated point light was considered, and then the convolution method [34] was used to obtain the simulated 480 nm light beam propagation. Briefly, by use of a random number evenly distributed between 0 and 1, the relationship was determined among the random number and a launch radius following a Gaussian probability density function with 1/e intensity radius. To account for the diverging source, defined by the numerical aperture (NA), the azimuthal angle is determined by a random number uniformly distributed from 0 to 2π and the elevation is determined from another random number distributed uniformly from 0 to sin-1(NA). [35]. The effect of intensity (number of photons), NA, and beam size was evaluated. A two-layer mouse brain model with optical properties [36] and depths is listed in S1 Table.

### Mouse preparation and optical stimulation setup

Adult (25–30g) male mice (B6.Cg-Tg, transgenic with Thy1-ChR2-YFP and B6129PF2/J as control, Jackson Laboratory) were used in this study (total = 20, n = 10 per group). The transgenic mouse strain B6.Cg-Tg(Thy1-COP4/EYFP)9Gfng/J (Jackson Laboratory) express ChR2 fused to YFP under the control of the mouse thymus cell antigen 1 (Thy1) promoter. Expression of the transgenic ChR2-YFP fusion protein is detected throughout the brain, including in the cortex, hippocampus, thalamus, midbrain, brainstem, cerebellar mossy fibers and retinal ganglion cells. Mice were maintained on a 12:12 light cycle (lights on at 07:00). Anesthetized animals (90 mg/kg ketamine, 10 mg/Kg xylazine) were placed in a stereotaxic frame (Kopf Instruments Inc., USA). A midline scalp incision was made and a 1.5 mm diameter craniotomy was performed. A guide cannula (Plastics One Inc., USA) with outer diameter of 1mm was unilaterally implanted in the right hemisphere using stereotaxic coordinates (AP: +1.0 mm; L: 0.8 mm; DV: 2.2 mm from bregma, angle of 20°) to allow for insertion of the optical fiber during the experiment. The guide cannula was secured to the skull using 2 anchor screws and cranioplastic cement (Plastics One Inc., USA). Body weight and possible signs of illness were monitored until recovery from surgery (∼ 1 week).

### In vivo electrophysiological recordings

Simultaneous optical stimulation of the ACC and electrical recording in either the ACC or the thalamus was carried out. Animals were deeply anesthetized with 90 mg/kg ketamine and 10 mg/kg xylazine. After aligning the mouse stereotaxically and surgically removing approximately 3mm^2^ of the skull dorsal to ACC and thalamus, a 1MΩ, 75μm-diameter tungsten electrode was stereotactically inserted into the craniotomized brain region (in mm: AP +1.0, L 0.6 and DV 2.0 for ACC or AP -2.0, L 2.0 and DV 3.5 for thalamus). For measurement of mechanical stimulation responses, two mechanical stimuli of increasing intensity (brush and pinch) were applied to the hind paw of the mice after insertion of the fiber and electrode. Each stimulus was applied once for 10 s, with an inter-stimulus interval of 20 s. The response to each mechanical stimulus was measured as the number of action potentials per second [9]. Recorded signals were bandpass filtered between 300 Hz and 8000 Hz, AC amplified either 5000x or 10000x (OmniPlex, Plexon Inc) and recorded using Plexcontrol software (Plexon Inc., USA). Plexon Offline Sorting and NeuroExplorer were used for analysis. Light pulses were generated by a function generator and synchronized with the OmniPlex recording system. Light power was measured using a standard light power meter (PM100D, Thorlabs Inc., USA) and adjusted so as to achieve power densities between 1 and 10 mW/mm^2^ at the fiber tip.

### Formalin test

Each mouse was randomly assigned to receive light stimulation or sham stimulation. Therefore the mice were allocated to one of four groups (1: Wildtype (WT) mice without light treatment; 2: WT mice with light stimulation; 3: control transgenic mice without light treatment; and 4: transgenic mice with light stimulation). Approximately 7 days after implanting the cannula, mice were lightly anesthetized with isoflurane and an optical fiber (Core: 200 μm and Cladding: 250 μm), adapted with an internal cannula, was inserted into the guide cannula. A baseline test was performed in which all mice received a 20 μL subcutaneous injection of normal saline (NS) into one hind paw. After the injection, light stimulation (or sham stimulation) was applied for 45 min. The time the mice spent on lifting and licking the paw was calculated and the pain sensation was scored as follows: paw lifting as 1 and paw licking as 2. After observation, the optical fiber was removed and mice returned to their cages.

Two days after establishing the baseline, the formalin test [37] was performed and involved the same mice tested individually in an observation chamber (30 cm x 12 cm x 30 cm) for the 45 min test period. As with the pre-formalin test, the mice were lightly anesthetized with isoflurane and an optical fiber was inserted into the guide cannula. Animals were then administered a 20μL subcutaneous injection of 1% formalin into the dorsal hind paw of the mouse using a micro-syringe with a 26-gauge needle. The light stimulation (or sham stimulation) was then applied and the amount of time the animal spent lifting or licking the injected paw was recorded during the 45 minute test period. Behavioral testing was performed by an observer unaware of the treatment in each experiment. For the groups receiving light stimulation, an optical fiber coupled with 473 nm laser was used during the 45 min test period. The intensity of fiber was set at 10 mW/mm^2^. The light flash continued for 20 ms during each stimulation cycle and repetition rate of 10 Hz. In all experiments attention was paid to the ethical guidelines for investigations of experimental pain in conscious animals.

### Histological Staining and Light Microscopic Analysis

Following behavioral and electrophysiological experiments, mice were deeply anesthetized with pentobarbital and perfused transcardially with phosphate buffered saline (PBS) followed by 4% paraformaldehyde (PFA) in PBS. Brains were removed carefully and post-fixed in 4% PFA for an additional 24 to 48 hrs, and subsequently transferred to 30% sucrose for 48 to 72 hrs. The tissue was embedded in Tek OCT (Sakura Finetek USA Inc., Torrence, CA) ans sliced at 40 μm sections of the ACC on a cryostat. Some slices were stained using 1% Thionin. Slices were then washed and mounted on gelatin-coated slides and stained with 0.25% Thionine stain (Invitrogen Inc, USA). Slices were then treated with mounting media (Fisher Scientific Inc., USA), and coverslipped. The location of the guide cannula was examined “blind” to behavioral outcome and group designation for all mice using an inverted microscope with a 2.5X objective.

### Immunohistochemistry

Fixed brain sections were sliced at 10 μm sections, washed multiple times using 0.1 M PBS solution and blocking solution (4% Donkey Serum, 0.5% Triton X-100) prepared in PBS was added and allowed to incubate at room temperature for 1 hr. Primary antibodies1:250, rabbit anti-GFP (Abcam, USA) and goat anti-GAD1 (Abgent Inc., USA) were made up in blocking solution then added and allowed to incubate overnight at 4°C. This was followed by three washes with washing solution (0.5% Triton X-100 in 0.1 M PBS) followed by 1 hour incubation with washing solution containing the secondary antibodies- Donkey anti-rabbit 488 (Jackson ImmunoResearch Laboratories, Inc., West Grove, PA) against anti-GFP, and Donkey anti-goat 594 (Jackson ImmunoResearch Laboratories, Inc., West Grove, PA) against GAD1. The sections were then washed thrice with washing solution and stained with 4',6-diamidino-2-phenylindole (DAPI) (Invitrogen, Carlsbad, CA) at room temperature for 10 min. Three more washes with washing solution followed by a final wash in 0.1 M PBS were performed. Lastly, there sections were affixed with glass coverslip using immunomount and imaged using a Zeiss epifluorescence microscope.

### Immunostaining

After obtaining cryostat sections, free-floating tissue was washed in PBS and then incubated for 30 min in 0.25% Triton X-100 and 1% bovine serum albumin (BSA) for 10 minutes. Slices were then washed and mounted on gelatin-coated slides, treated with fluorescent-mounting media, and coverslipped. Primary antibody incubation was performed 1 h at room temperature in 1% BSA/PBS (goat anti-GAD1 1:200, Abgent Inc., USA). Sections were then washed and incubated with secondary antibody (Rabbit anti-goat, FITC 1:500, Invitrogen Inc.) and Rabbit anti-GFP conjugated with Alexa 555 (Invitrogen Inc., USA) for 1 h at room temperature. Following 10 min incubation with Hoechst (1μg/mL), sections were washed and mounted on microscope slides with mounting media. Expression of ChR2-EYFP and GAD1 were then examined using a Zeiss laser-scanning confocal microscope with a 40X objective. Mice showing no EYFP expression in the ACC or mice showing cannula or fiber placements outside of the ACC were excluded from analysis.

### Statistics

SPSS (IBM) were used to analyze data. The data were plotted as mean ± S. D. Statistical significant differences were identified by one-way analysis of variance (ANOVA) followed by Tukey’s post-hoc tests. *p*<0.05 was considered statistically significant.

## Results

### Inhibitory ACC neurons express ChR2

**Fig 1:**
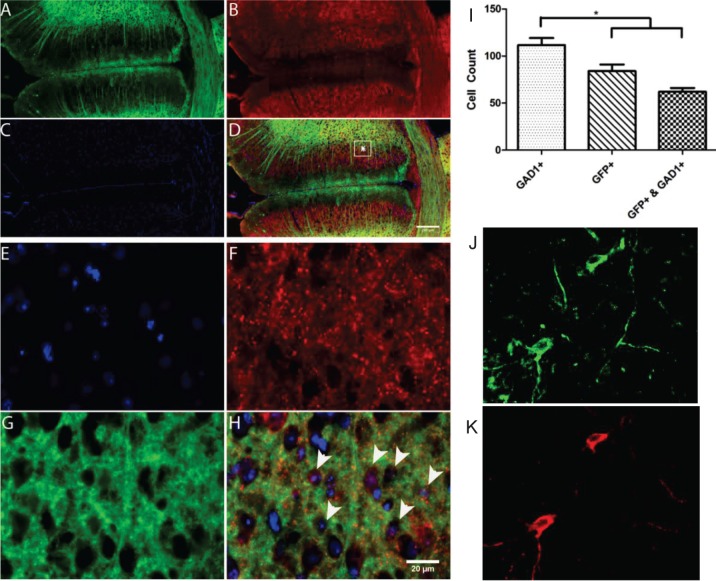
GAD1 and GFP positive labeled cells in the anterior cingulate cortex. A: GFP, B: GAD1, C: DAPI, D: GFP+ labeled cells overlaid with GAD1 and DAPI. High magnification image at the anterior cingulate cortex (E: DAPI, F: GAD1, G: GFP, H: co-expression). (I) Number of cells expressing GFP+, GAD1+, or GFP & GAD1 in the ACC. Confocal images of immunostaining of the ACC neurons with GAD1-FITC (J), and YFP-Alexa 555 (K). Scale bar: 50 μm.

To examine the expression of ChR2 in specific cell types, we immunostained the ACC region in transgenic mice with cell-specific antibodies, along with the GFP antibody. Fig. 1 shows GAD1 and GFP positive labeled cells in the anterior cingulate cortex. GAD1 is known to be expressed in GABAergic neurons. Co-expression (Fig. 1D) revealed that in the ACC region, ChR2 is expressed in inhibitory neurons in the transgenic mice. Fig. 1 E-H shows high magnification image at the ACC showing co-expression of GFP and GAD1 cells. Fig. 1I shows that significant number of inhibitory neurons express ChR2 (GFP+) in the ACC and 80% of neurons expressing ChR2 in the ACC are found to be inhibitory. Fig. 1 J & K show confocal images of neurons in ACC immunostained with GAD1-FITC and YFP-Alexa 555 respectively. Z-stack composite images of two different regions in ACC, immunostained with GAD1-FITC (green) and YFP-Alexa 555 (red) are shown in S1 Fig.

### Effect of laser intensity and frequency on ACC activity

**Fig 2:**
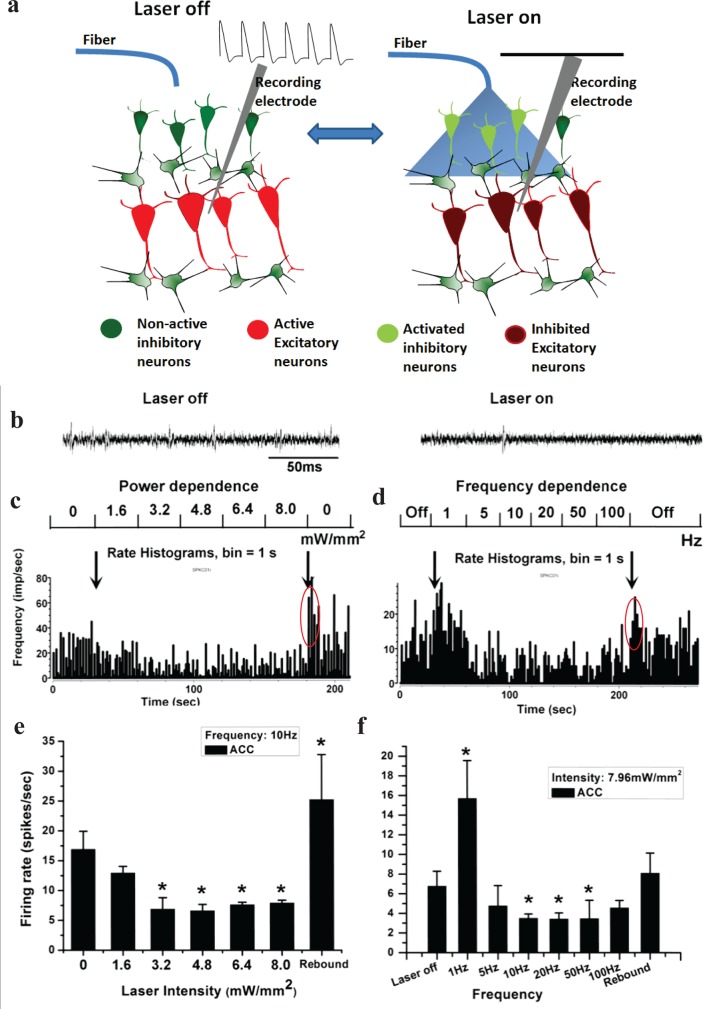
*In-vivo* Electrophysiology of inhibition of ACC neurons by optogenetics. (a) Schematic showing the mechanism of post-synaptic silencing of excitatory neurons due to optogenetic-stimulation of inhibitory neurons in ACC. (b) Representative neuronal firing in ChR2 transgenic mouse without (left) and with (right) light stimulation for 250 ms. (c) Rate histogram of neuronal spikes with varying intensity of light stimulation in the ACC. (d) Rate histogram of neuronal spikes with varying frequencies of light stimulation in the ACC. (e) Intensity-dependent firing rate of neurons in the ACC. (f) Frequency-dependent firing rate of ACC neurons. Rebound activity of excitatory neurons after switching off the laser is encircled. *P < 0.05 vs. control (laser off).

To verify post-synaptic inhibition in ACC as the mechanism by which light activation of GAD-1+ neurons may suppress pain perception, electrophysiological recordings were made from spontaneous spiking activity of ACC, without peripheral stimulation (Fig. 2A). Also, light intensity and frequency of stimulation was varied to determine effect of stimulation parameters for efficient modulation of neural activities of the ACC. As shown in Fig. 2B, the spiking of ACC neurons decreased with light stimulation (on) as compared to the off-condition. The effect of various light intensities on spiking rate (Fig. 2C and E) showed that excitatory neurons of ACC could be silenced (*P < 0.05 vs. laser off) likely by stimulating the inhibitory inter-neurons at 10 Hz. The threshold saturation laser intensity was observed to be ∼3.5 mW/mm^2^. S2A and C Fig. shows the inter-spike interval (ISI) as a function of intensity corresponding to Fig. 2C and 2E respectively. The mean ISI (S2C Fig.) was found to increase as a function of laser intensity and saturate at ∼3.0 mW/mm^2^ implying efficient laser-induced silencing of depolarizing ACC neurons.

Switching off the laser beam, we believe suppresses excitatory ACC neuronal activity leading to rebound activity [38,39] (*P < 0.05 vs. laser off, n = 3) in ACC. This further suggests that the laser is stimulating inhibitory neurons. Following removal of the laser, the duration of rebound burst firing was ∼10 sec, after which the firing was still higher than the spontaneous activity. In S3A Fig., we show rebound burst firing rate of ACC depolalirizing neurons (spike rates ∼ 5 times that during laser stimulation and ∼2 times the spontaneous firing rate) after switching off the laser stimulation (8 mW/mm^2^, 10 Hz). Increasing the frequency to 100Hz, while keeping the intensity same, led to rebound burst firing rate ∼ 3 times that observed during laser stimulation, which is ∼1.6 times the spontaneous firing rate (S3B Fig.). This observation is consistent with the optical activation of inhibitory ACC neurons.

Frequency dependent response of ACC-neurons (Fig. 2D and F) showed surprising results. The firing rate of ACC depolarizing neurons reached its maximum for 1Hz laser stimulation as compared to the laser-off condition (*P < 0.05 vs. laser off, n = 3). However, stimulation of inhibitory neurons in the ACC over a broad frequency range (5 to 100 Hz) led to a significantly decreased (*P < 0.05 vs. laser off, n = 3) firing rate of spiking ACC-neurons (Fig. 2D and F). The optimal frequency range for reducing firing of excitatory neurons is observed to be in the 10–50 Hz. In this case also, the rebound activity (higher spike rates) in ACC after switching off the laser was observed. S2B and D Fig. show the inter-spike interval (ISI) as a function of stimulation frequency, corresponding to Fig. 2D & F respectively. While the mean ISI (S2D Fig.) was found to increase as a function of laser frequency, the ISI was at its maximum at 10–50 Hz stimulation (S2D Fig.), implying efficient laser-induced silencing of excitatory ACC neurons. This can be seen in the frequency-dependent raster of neuronal firing in ACC (S2E Fig.).

### Simulation of light propagation in ACC

To deliver stimulation light (blue) in ACC, an optical fiber probe (S4A Fig.) was inserted through the cannula implanted in the mice. Monte Carlo (MC) simulations were conducted in order to determine the required light intensity to be delivered for stimulating neurons in ACC, based on the location of the fiber tip. The 480 nm photon fluence distribution obtained from MC simulation in a two-layered cortex for 10^6^ incident photons (NA = 0.22) is shown in S4B Fig. The attenuation of intensity of light of particular wavelength was simulated using absorption and scattering properties (μ_a_: absorption coefficient; μ_s_: scattering coefficient) of the brain tissue at that wavelength. The incident light intensity (I_0_) in tissue (I) is known to decay as a function of tissue depth or thickness $\left(\text{t}):\;\text{I}={\text{I}}_{0}\;[{\text{e}}^{\;-\;\mu_{\text{eff}}\;\text{t}}\right]$, where μ_eff_, the effective attenuation coefficient is given by sqrt [3μ_a_(μ_a_+μ_s_’)]; and μ_s_’ is the reduced scattering coefficient, given by μ_s_(1-g), g being the anisotropy factor of the forward scattering tissue. S4C Fig. shows theoretically calculated variation of light intensity (I) as a function of brain depth (t) for known absorption and scattering properties at 480 nm.

### Modulation of pain behavior by driving inhibitory network of ACC with light

Based on the MC simulation, the known threshold for ChR2-stimulation and the electrophysiological measurements, the power of the incident light was selected for *in-vivo* behavioral modulation study. The intensity threshold of ∼3 mW/mm^2^ was observed to generate lowest spike rate in ACC neurons (Fig. 2C, E). At a depth of 0.8 mm (extent of ACC), the blue laser beam gets attenuated by 90% (S4C Fig.). Therefore, incident laser intensity of 10 mW/mm^2^ was used for behavioral studies so that at depth of 0.8 mm, the effective light intensity is ∼ 1 mW/mm^2^, which is above known threshold for optogenetic stimulation. This illumination intensity corresponds to an activation volume of ∼ 0.6 mm^3^ of the ACC (cylinder marked in S4B Fig.). Further, though a range of frequency (10–50 Hz) was found to be optimal for inhibiting activity of ACC neurons (Fig. 2D, F), to minimize heating during behavioral studies 10 Hz (20 ms pulse) was used. For behavioral modulation of pain by optogenetic stimulation of ACC, the optical fiber was positioned exactly above the ACC region (S5A Fig.), via a guide cannula which was unilaterally implanted using standard stereotaxic techniques, into the right hemisphere. Histological (S5A Fig.) and fluorescence (S5B Fig.) imaging of the brain slices shows expression of ChR2-YFP at the distal end of the implanted cannula.

**Fig 3:**
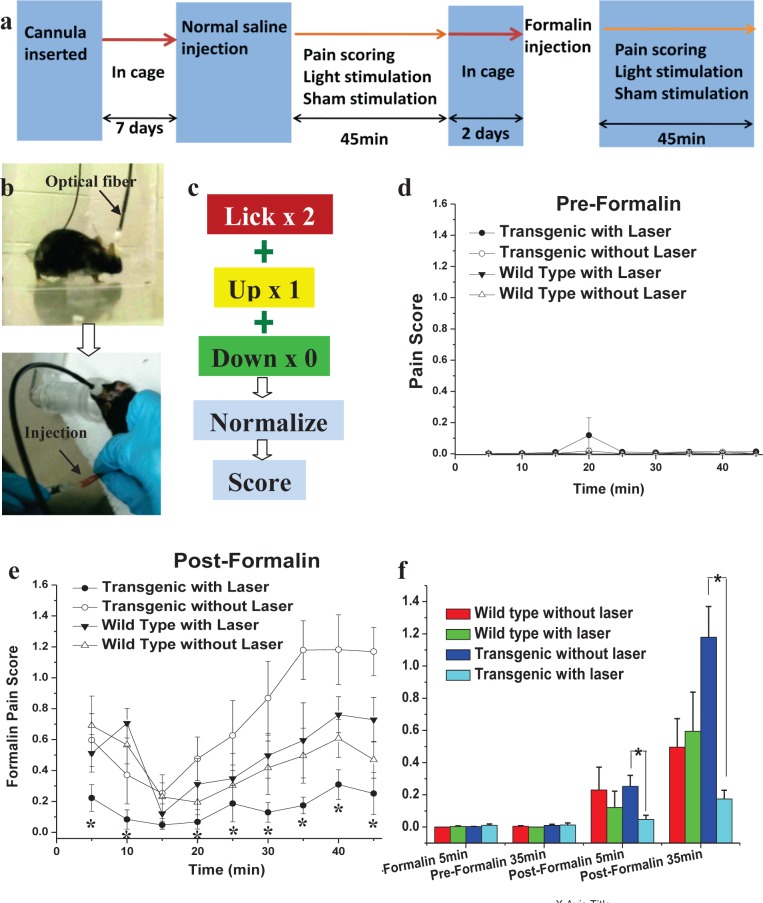
Behavioral assay of pain inhibition by optogenetic modulation of ACC. (a) Experimental scheme for Formalin test. (b) Optical fiber mounted via cannula in mouse brain (upper panel). Lower panel shows saline or Formalin injection in hind paw. (c) Pain scoring. (d) Pre-formalin pain score of different mice groups, (e) pain score of same groups of mice in post-formalin condition. *P < 0.05 vs. others (transgenic laser off, wild type laser on and wild type laser off). (f) Histogram for comparison of pre and post formalin pain scores for various mice groups at two different time points. *P < 0.05 vs. control (laser off), n = 10 for each group.

To evaluate whether or not optogenetic stimulation of inhibitory neurons in the ACC would modulate pain behavior, the formalin test assay of acute inflammatory pain [37] was conducted 1 wk after implantation of the cannula, as shown in Fig. 3A. The formalin test is a widely used tonic model of continuous pain involving neurogenic, inflammatory, and central mechanisms of nociception. Both transgenic and wild type mice were randomly assigned to receive either sham stimulation or laser stimulation. A pre-formalin test was performed (S1 Movie) and involved fiber optic insertion into the guide cannula followed by a subcutaneous injection of normal saline (NS) to the dorsal surface of one hind paw (Fig. 3B). Application of the light stimulus through the optical fiber in pre-formalin tests (S2 Movie) did not lead to any significant behavioral change. Two days after the baseline test, the formalin test was performed in mice that were individually placed into an observation chamber for 45 min. After the formalin injection, the sham (S3 Movie) or light stimulation (S4 Movie) was applied, and the pain response in the form of lifting or licking the injected paw was recorded and scored as shown in Fig. 3C.

The pain scores in all pre-formalin groups were found to be lower than 0.2 (Fig. 3D), and no significant difference was observed among the groups without and with laser stimulation. This confirms that the light stimulation does not modulate behavior in the absence of noxious pain stimuli. As expected, the pain scores following the formalin injection (Fig. 3E) were higher than the scores in pre-formalin test (Fig. 3D). Consistent with previous formalin-test studies [37], a typical biphasic behavioral response was observed. There was an initial 5–10 min period of activity in response to pain, followed by a short period of diminished responding, and then a gradual return to normal during the remainder of the 40-min test period. The pain scores in wild type mice did not change significantly with laser stimulation from the non-stimulated control condition. This indicates that the laser stimulation did not affect the pain sensation in wild type mice without ChR2.

Of primary interest is that formalin induced less pain in transgenic mice with laser stimulation (S4 Movie) scoring 0.22 ± 0.08 (n = 6), significantly lower than that observed for the wild type group (0.69 ± 0.19, n = 7). Switching off the laser stimulation led to increased pain sensation in transgenic mice (S3 Movie). Surprisingly, 20–45 min after formalin injection, the pain scores in transgenic mice without laser stimulation (n = 6) was found to be higher than wild type mice (p <0.05). This indicates that the transgenic mice are more sensitive to pain than wild type mice. However, the reason behind the slightly higher sensitivity of pain in ChR2 expressing animals is still unknown and requires further investigation.

It is important to note that the laser stimulation of inhibitory neurons of ACC expressing ChR2 in freely moving transgenic mice caused a dramatic and significant inhibition of pain (Fig. 3E). The histogram for comparison of pre- and post-formalin pain scores for various mice groups at the two different test periods is shown in Fig. 3F. The dramatic inhibition of pain behavior due to optogenetic stimulation of ACC (expressing ChR2 in inhibitory neurons) may be attributed to the post-synaptic inhibition of excitatory neurons (Fig. 2A).

### Optogenetic stimulation of ACC inhibits thalamic activity

**Fig 4:**
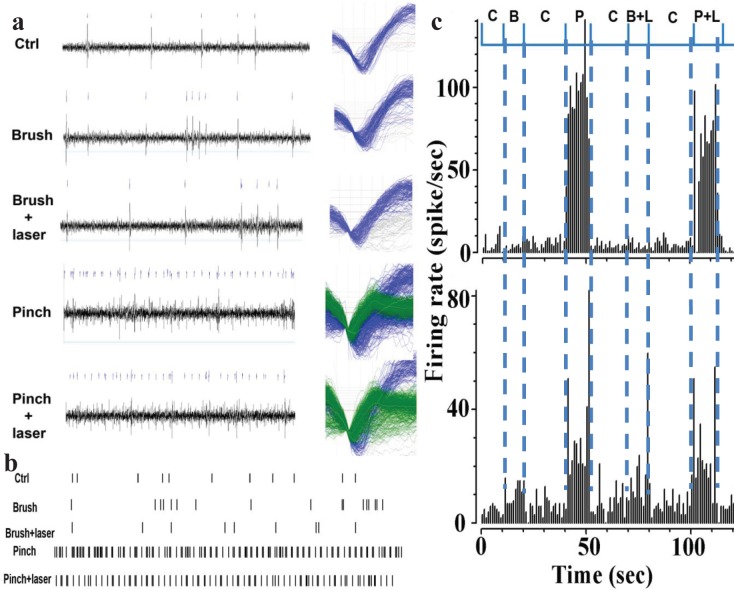
Modulation of neuronal activities in the thalamus in response to brush and pinch. (a) Representative raw data of spiking activity (left) and waveforms (right). The waveforms of different firing units are grouped as blue and green colors. (b) Raster scan. (c) Response of different neuronal groups in the thalamus due to pinch and brush. C: Laser off (control), P: Pinch, B: Brush, L: Laser on, P+L: Pinch + Laser on, B+L: Brush + Laser on.

To examine the ascending pathway of pain inhibition, in addition to recordings in ACC (Fig. 2) during optogenetic stimulation of ACC, electrophysiological recording of neuronal activities in the ventral posterior complex (VPL/VPM) area of thalamus was carried out, as this region in thalamus were made from is known to be involved in pain processing pathway. Location of optical fiber (L: 0.6 mm; DV: 2 mm, angle of 20°) in ACC is shown in H&E stained, coronal section (AP: +1.0 mm, from bregma) of mouse brain (S5C Fig.). As shown in S5D Fig., upon optogenetic stimulation of ACC, the electrophysiological recordings in VPL/VPM of thalamus was carried out for a range of electrode location(s) (L: 2 ± 0.2 mm; DV: 3.5 ± 0.2 mm). The responses to pinch and brush mechanical stimuli in transgenic mice were recorded in different neuron groups in the thalamus subsequent to stimulation of the ChR2 expressing inhibitory neurons in ACC. Fig. 4A shows the representative raw data of spiking activity (left) and waveforms (right) for brush and pinch stimuli with and without laser. The two distinct firing units, sorted by use of principal component analysis using the Plexon offline sorter, are shown as color-coded. The effect of optogenetic stimulation of ACC-inhibitory neurons on neurons in thalamus can be seen in the representative raster scan of control, brush, and pinch (Fig. 4B). In Fig. 4C, we show the histogram of the two distinct firing units in the thalamus due to pinch and brush. The optogenetic-reduction in the responses evoked due to pinch was found to be greater than that due to brush (Fig. 4C). While changes in spike rate due to pinch are higher than the control (laser off) in first unit (upper panel of Fig. 4C), that due to brush is higher in the second unit (upper panel of Fig. 4 C). While both groups of neurons were sensitive to pinch (noxious stimuli), laser stimulation significantly decreased (n = 5) the firing responses to pinch in group 1 (upper panel of Fig. 4C). The pinch-response in group 2 neurons (lower panel of Fig. 4C) was affected to a lesser extent by optogenetic stimulation. Thus, optogenetic modulation can lead to pain inhibition by selectively enhancing the inhibition circuitries of ACC-PAG-Dorsal horn and ACC-Thalamus-PAG-Dorsal horn [9,40].

## Discussion

Despite the progress achieved over many years, many forms of chronic pain are still resistant to conventional treatments. Here, we investigated the optical stimulation of inhibitory neural networks in the anterior cingulate cortex, a brain region mediating affective responses to noxious stimuli [41,42]. Electrical response of the ACC to peripheral stimulation successfully correlated with S1 neuronal activity, and inhibition of ACC activity alleviated the mechanical allodynia [43]. Unlike electrical stimulation, we demonstrated that specific neurons in ACC can be activated with physiologically relevant temporal precision. Since ChR2 is expressed in GABAergic neurons in ACC (Fig. 1, S1 Fig.), optogenetic activation of ACC activates those inhibitory neurons, and therefore reducing total neuronal activity in the ACC (Fig. 2 and S2 Fig.). It has been shown recently that nerve injury results in potentiation of the intrinsic excitability of ACC pyramidal neurons, whereas the cellular properties of interneurons were unchanged [44]. We demonstrated that activation of GABAergic ACC interneurons reduces pain perception. Balance between cortical excitation and inhibition is essential for normal brain function and signal processing. Activation of inhibitory interneurons in the cortex by transcranial magnetic stimulation have analgesic effects [45] [46]. Furthermore, changes in GABA content have been reported in the thalamus, anterior insula and ACC of chronic pain patients [47]. Thus, modulation of inhibitory connectivity in the ACC has a profound effect on pain perception. While we cannot exclude the activation of glutamatergic neurons in our study, a recent model on the ACC network reported the ratio of excitatory and inhibitory cells to be 0.8 [44]. Thus the majority of the ACC neurons seem to be inhibitory.

The electrophysiological data showed interesting intensity and frequency dependence (Figs. 2, 4; and S2 and S3 Figs.), our behavior experiments (Fig. 3), conducted at saturated laser power levels with 10 Hz stimulation show a robust reduction of pain behavior. However, it will be valuable to compare pain-inhibition efficacy of two approaches, namely (i) activation of inhibitory neurons vs (ii) inhibition of excitatory neurons in the ACC. It will be especially interesting to optogenetically control the excitation to inhibition ratio in the ACC by targeting glutamatergic (excitatory) and GABAergic (inhibitory) neurons [48]. Understanding of electrical activity of specific cell types within the ACC using combinatorial optogenetic means will provide significant insights into chronic pain processing and may lead to new therapeutic strategies. It is important to note that, although GABAergic neurons are described conventionally as participators only in local connectivity of cortical function, a subset of phylogenetically-conserved cortical-GABAergic neurons were found [49,50] to project axons across long distances. We believe the optogenetic stimulation approach, combined with pharmacology, multi-site electrophysiology, and behavioral assays will decipher the role of these newly identified [49,50] long-range cortical GABAergic interneurons in pain inhibition. In order to further dissect the descending pathway of pain inhibition by optogenetic modulation, neuronal activities from ACC, thalamus, and dorsal horn can be simultaneously recorded using multiple electrodes. The optogenetic approach can foster understanding of neural circuitry within the ACC and other areas of brain, thus providing significant insights into chronic pain processing. The findings presented in this paper have translational potential by replacing the single photon laser and optical fiber (used in this study) with an implantable μLED [51,52,53] or non-invasive two-photon beam [54].

## Conclusions

To conclude, our results clearly demonstrate, for the first time, that optogenetic stimulation of inhibitory neurons in ACC leads to decreased neuronal activity in ACC, and a dramatic reduction of pain behavior in a mouse model of pain. A distinct, frequency-dependent inhibition of spiking activity in the ACC was observed, which implies that modulation of inhibitory circuitry of the ACC, using specific frequency of light, can lead to significant behavioral pain modulation. Moreover, our results showed that the electrophysiological responses of different neuronal units (in the thalamus) to different types of pain (formalin injection, pinch and brush) are modulated to a different extent subsequent to stimulation of ACC expressing ChR2 in inhibitory neurons. Taken together, optogenetic stimulation is believed to be less invasive and highly specific for pain inhibition as compared to its electrical stimulation counterpart. Mechanistic study using cell-specific excitation of targeted neural circuitry in the ACC by combinatorial optogenetic means can lead to better understanding of the functioning of the pain processing neuronal network in ACC. These results open up a new avenue to clinically inhibit chronic pain by modulating specific neural circuitry of the ACC and also lead to development of therapeutic drugs targeting this pathway.

## Supporting Information

S1 Table Optical properties of the two-layered mouse brain model used for Monte Carlo simulation. (EPS)

S1 Fig Confocal z-stack imaging of Immunostained ACC neurons. Z-stack composite images of two different regions (a & b) in ACC, immunostained with GAD1-FITC (green) and YFP-Alexa 555 (red). Scale bar: (a) 50 μm, (b) 20 μm.
(EPS)

S2 Fig *In-vivo* Electrophysiology shows inhibition of neural activity in ACC by optogenetic stimulation. (a) Inter-spike interval (ISI) with varying intensity of light stimulation in the ACC. (b) ISI of neuronal spikes with varying frequencies of light stimulation in the ACC. (c) Intensity-dependence of ISI of neurons in the ACC. (d) Frequency dependence of ISI in the ACC. (e) Raster of neuronal firing in the ACC at different stimulation frequencies.
(EPS)

S3 Fig Rebound burst firing rate of ACC-excitatory neurons after switching off the laser stimulation (8 mW/mm^2^) at (a) 10 Hz, and (b) 100 Hz. The bars around mean represent standard error of means (n = 5).
(EPS)

S4 Fig Optimization of parameters for optogenetic stimulation of ACC. (a) Fiber probe outside cannula, (b) Monte Carlo simulation of 480 nm light propagation in two-layered cortex for 10^6^ incident photons (NA = 0.22). (c) Calculated attenuation of intensity as a function of tissue depth or thickness. The tissue optical properties of white matter at 480 nm, used for the simulation, are listed in inset.
(EPS)

S5 Fig Histology of optogenetic stimulation and electrophysiological detection of neural activities in ACC and Thalamus. (a) Histological staining showing a representative example of the position of cannula. (b) Confocal fluorescence imaging of ChR2-YFP expression in transgenic mouse brain near the inserted cannula. Scale bars: 400 μm in c, and 40 μm in d. (c) H&E stained, coronal section of mouse brain at (AP: +1.0 mm, from bregma). Location of optical fiber (dotted white line, L: 0.6 mm; DV: 2 mm, angle of 20°) to stimulate ACC (white circle) and electrode (dotted black line) to record electrical activities in ACC upon optogenetic stimulation. (d) H&E stained, coronal section of mouse brain at (AP: -2.0 mm, from bregma). Range of electrode location(s) (dotted black lines, L: 2 mm; DV: 3.5 mm) to record electrical activities in Thalamic region (marked by white circle) upon optogenetic stimulation of ACC.
(EPS)

S1 Movie Pre-formalin behavior of a representative transgenic mouse without laser stimulation. (WMV)

S2 Movie Pre-formalin behavior of a representative transgenic mouse with laser stimulation. (WMV)

S3 Movie Post-formalin behavior of a representative transgenic mouse without laser stimulation. (WMV)

S4 Movie Post-formalin behavior of a representative transgenic mouse with laser stimulation. (WMV)
